# Covert spatial selection in primate basal ganglia

**DOI:** 10.1371/journal.pbio.2005930

**Published:** 2018-10-26

**Authors:** Fabrice Arcizet, Richard J. Krauzlis

**Affiliations:** Laboratory of Sensorimotor Research, National Eye Institute, Bethesda, Maryland, United States of America; UCSF Sandler Neurosciences Center, United States of America

## Abstract

The basal ganglia are important for action selection. They are also implicated in perceptual and cognitive functions that seem far removed from motor control. Here, we tested whether the role of the basal ganglia in selection extends to nonmotor aspects of behavior by recording neuronal activity in the caudate nucleus while animals performed a covert spatial attention task. We found that caudate neurons strongly select the spatial location of the relevant stimulus throughout the task even in the absence of any overt action. This spatially selective activity was dependent on task and visual conditions and could be dissociated from goal-directed actions. Caudate activity was also sufficient to correctly identify every epoch in the covert attention task. These results provide a novel perspective on mechanisms of attention by demonstrating that the basal ganglia are involved in spatial selection and tracking of behavioral states even in the absence of overt orienting movements.

## Author summary

The ability to respond selectively to sensory inputs is a crucial brain function, one that is implicated in variety of high-level brain disorders. The basal ganglia are a set of evolutionarily ancient structures best known for their role in controlling motor actions and more recently implicated in nonmotor cognitive functions. Here we show for the first time, to our knowledge, that neuronal activity in the caudate nucleus, one of the major input structures of the basal ganglia, is modulated during a covert selective attention task. Two monkeys were trained to detect a subtle change in a visual motion stimulus at a cued location while ignoring a similar stimulus at a second, uncued location. Monkeys were not allowed to look directly at the stimuli, but they monitored them covertly and released a joystick to report their choices. We found that caudate neurons strongly selected the spatial location of the relevant stimulus throughout the task even in the absence of any overt movement. This spatially selective activity was dependent on task and visual conditions and could be dissociated from goal-directed actions. These results advance our understanding of the basal ganglia and provide a novel perspective on the brain circuits for selective attention.

## Introduction

The basal ganglia are known to govern behavior by disinhibiting desired actions and inhibiting undesired actions [1]. The basal ganglia have also been implicated in perceptual and cognitive functions [2], such as the encoding of object values [3] and action values [4], and signals related to visual decision-making [5,6]. Neurons in caudate nucleus, one of the major input structures of the basal ganglia, display a number of decision-related signals as monkeys formulate their perceptual choice (reported by a saccadic eye movement) during a visual motion discrimination task [5], consistent with a possible role for the basal ganglia in regulating the use of sensory evidence. Computational studies have provided a general framework that might account for these diverse functions, suggesting that the basal ganglia act as an integration center that plays a crucial role in representing the subject’s “belief state" about the current context that helps constrain the process of action selection [7]. If this view of basal ganglia function is correct, then during tasks involving spatial attention—for example, selectively basing a perceptual decision on a stimulus at one location while actively ignoring a distracter stimulus at a different location—one might expect to find neuronal signals in the caudate related to spatial selection and the internal encoding of belief states, even when no overt action or goal-directed movement is required.

Spatial selection in the caudate nucleus has been studied principally during tasks requiring a goal-directed movement, either with the eyes [8] or arms [9]. In both cases, a subset of caudate neurons exhibits a degree of spatial selection as the monkey anticipates a movement instruction. Caudate neurons also show some spatial selection during the delay period preceding an action directed to the particular location [10]. In these paradigms, it is ambiguous whether the spatially selective activity is related to the visual location itself or to the spatial goal of the movement. In the antisaccade paradigm, which dissociates the visual target location from the movement endpoint, some caudate neurons have higher activity for antisaccades than for prosaccades [11,12]. However, even in this task, the instructional cue and movement endpoint are tightly linked, because antisaccades require a goal-directed eye movement to the location diametrically opposite the visual cue. Consequently, it is not known whether caudate activity can be spatially selective when animals attend covertly to a particular visual location that has no link to the end point of a goal-directed movement.

Another well-documented contribution of the basal ganglia in the primate is the coordination of motor outputs by grouping individual movements into action “chunks” [13] during sequences of eye movements [4] or arm movements [14]. A recent study in rats found that striatal neurons were activated sequentially throughout the course of the delay period when animals had to wait before making a response, suggesting that sequence-related activity in the striatum might be a component of spatial working memory [15]. These studies raise the possibility that sequence-related activity in the primate striatum might also apply to the successive behavioral states that subjects pass through during the performance of covert spatial selection tasks in the absence of goal-directed or orienting movements, but this possibility has not yet been directly tested.

To test whether the primate striatum plays a more general role in spatial selection in the absence of overt movements, we examined the activity of caudate neurons while monkeys performed a covert attention task. Animals were trained to covertly monitor a peripheral visual motion stimulus and report when the direction of motion changed by releasing a joystick; unlike previous studies, the task required spatial selection but did not include movements toward a spatial goal. Our results demonstrate that the primate striatum is involved in covert spatial selection by showing that (1) caudate neurons strongly discriminated the location of behaviorally relevant events, even though no goal-directed movement was involved during different crucial periods of the trial; (2) this spatially selective activity required the presence of a distracter and often disappeared when only a single visual stimulus was presented, indicating that the spatial selection was not only related to reward expectation; (3) caudate neurons often showed response-choice activity that also depended on the visual configuration; and (4) the pattern of activity across caudate neurons was sufficient to correctly identify epochs in the covert attention task. Our results illustrate a possible common thread between the motor and cognitive functions of the basal ganglia.

## Materials and methods

### Ethics statement

All procedures and animal care were approved by the National Eye Institute Animal Care and Use Committee and complied with the Public Health Service Policy on the humane care and use of laboratory animals.

### Animals

Data were collected from two adult monkeys (*Macaca mulatta*; Monkey R, 11 kg; Monkey P, 14 kg). Under isoflurane and aseptic conditions, we surgically implanted plastic headposts and recording chambers. In both animals, recording chambers (28 × 20 mm) were tilted laterally 35 degrees and aimed at the caudate head and body (20 mm anterior, 6 mm lateral).

### Experimental apparatus

The animals were seated in a primate chair (Crist Instrument, Hagerstown, MD, United States) with their head fixed inside a darkened booth. Animals were positioned 48 cm in front of a 100 Hz VIEWPixx display (VPixx Technologies, Saint-Bruno, QC, Canada), and experiments were controlled using a modified version of PLDAPS [16]. Eye position was monitored using an EyeLink 1000 infrared eye-tracking system (SR Research, Ottawa, Ontario, Canada). Animals reported their choices using a joystick while maintaining central fixation. Joystick movements were measured as changes in voltage from a single-axis joystick (CH Products, model HFX-10) mounted in the front wall of the primate chair and oriented so that the monkey deflected the joystick downward to initiate and continue each trial and released the joystick back to its central neutral position to indicate a response. Joystick release times (reaction times) were computed by detecting the onset of the step change in voltage from the joystick, similar to saccade detection.

**Fig 1:**
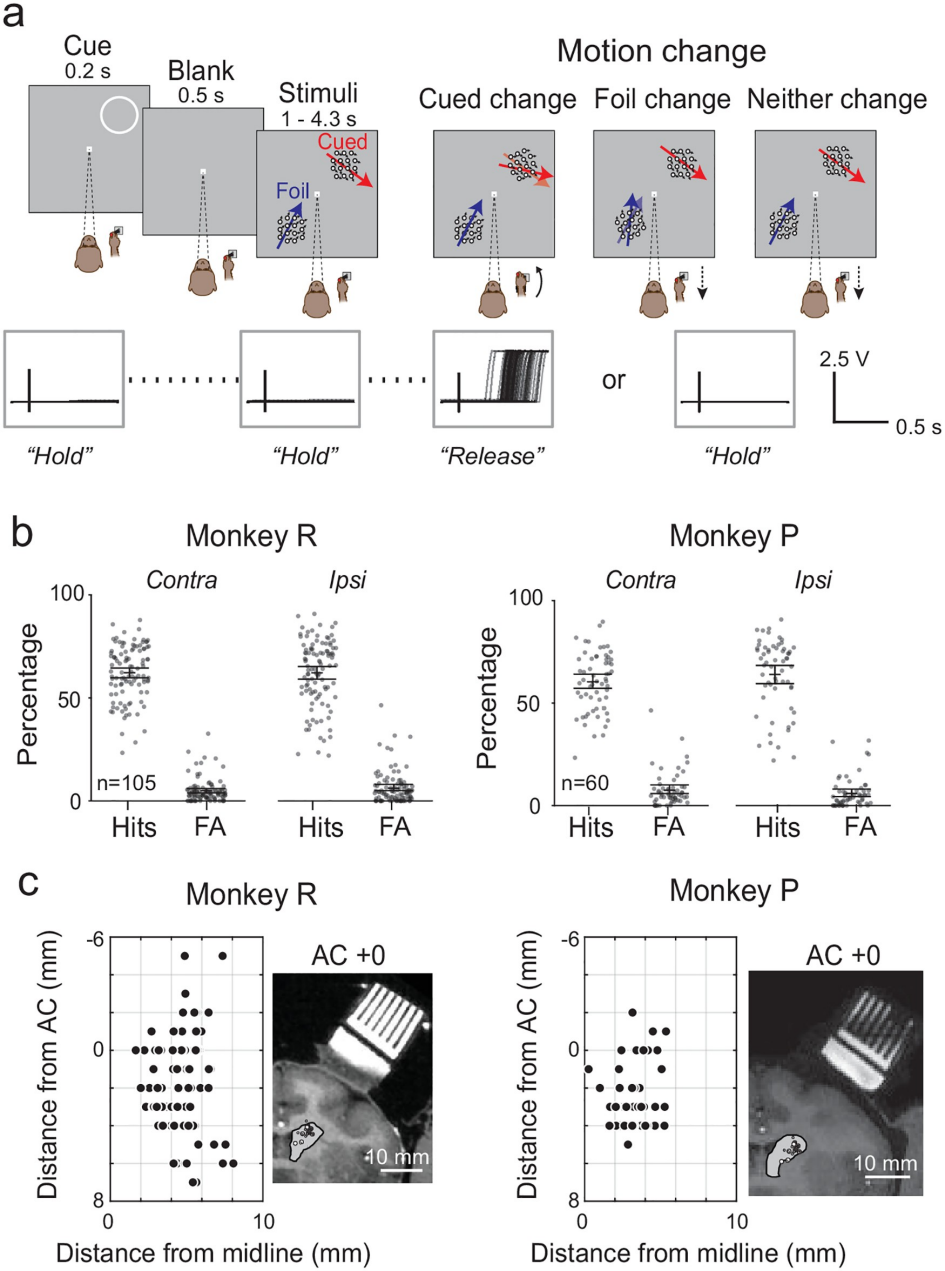
Change-detection task, behavioral performance, and recording sites. (A) Task sequence in the covert attention task. While fixating a central spot and pressing down a joystick, a peripheral spatial cue (ring) was flashed for 0.2 s to indicate which part of the visual field the monkey should monitor. After a blank of 0.5 s, two motion patches were presented: one at the location previously cued (cued location) and the other one diametrically opposed (foil location). The monkey should detect when the motion direction changed at the cued location by releasing the joystick and otherwise keep holding the joystick down if the motion direction changed at the foil location or if no change occurred. Inserts at the bottom show the joystick voltage traces for one experimental session aligned on cue onset, motion stimulus onset, and cue or foil change (from left to right, respectively; vertical lines show the zero). Black traces indicate the individual trial joystick voltages. (B) Behavioral performance in the task for both monkeys. Each dot represents the percentage of correct change trials (Hits) and FAs for a single behavioral session (*n* = 105 for Monkey R, *n* = 60 for Monkey P), separately for trials with the cue contralateral (“contra”) or ipsilateral (“ipsi”) to the site of recording. Error bars indicate 95% CIs of the mean. (C) Location of neuronal recording sites in the left caudate nucleus for both monkeys. Each dot represents one recording site. Positive values on the y-axis indicate locations anterior to the AC. MRI images represented the coronal section at the AC (AC+0). Dots represented the locations of the recorded neurons within the caudate nucleus (gray surfaces) for this coronal section. Scale bar indicate 10 mm. Underlying data available in S1 Data. AC, anterior commissure; FA, false alarm.

Neurons were recorded using tungsten in glass-coated electrodes with impedances of 1–2 MOhm (Alpha Omega, Alpharetta, GA, US). Electrode position was controlled with a stepping motor microdrive (NAN Instruments, Nazaret Illit, Israel). The electrical signal was amplified and filtered, and single-unit activity was recorded online using the Plexon MAP system spike sorting software (Plexon, Dallas, TX, US). Spike waveforms were analyzed again off-line to confirm that recordings were of single well-isolated neurons. We recorded neurons in the head and body of the left caudate nucleus for both animals with a range from anterior commissure (AC) AC+7 to AC−5 for Monkey R and AC+5 to AC−2 for Monkey P (with AC as anterior commissure at AP20, Fig 1C). Neurons were considered to be in caudate nucleus according to their location (based on MRI scans) and their low background activity at >10 mm below the dural surface. In this study, we recorded only from phasically active neurons (PANs), which we identified based on their low background activity compared to the tonic activity from the cholinergic interneurons (TANs). Single units with low or unstable firing rates across the session or with no task-related activity were excluded from the analysis.

### Memory-guided saccade (MGS) task and joystick task

Upon isolating spikes from a caudate neuron, we first tested neurons with an MGS task in most cases (179/227, 78%). The monkey fixated a central spot for 0.5 s, after which a spot stimulus was flashed for 0.15 s at one of eight possible peripheral locations. The monkey maintained fixation until the fixation point turned off, at which point the monkey made a saccade to the memorized cued location within 0.5 s in order to receive a reward. We defined four different periods to test whether activity was modulated: visual (0–0.5 s) and delay (0.5–1 s) periods aligned on stimulus onset, saccade (−0.2:0.1 s) and postsaccadic (0.1:0.5 s) periods aligned on saccade onset. A total of 60% (108/179) of caudate neurons tested with MGSs were significantly modulated for at least one of these four temporal periods (Wilcoxon rank sum test, *p* < 0.05 comparisons with baseline activity [−0.4:0 s]), with the majority showing a preference for the contralateral side during the visual (55%) and saccade (57%) periods.

Because not all caudate neurons were modulated during MGSs, we also tested most neurons with a joystick task (190/227, 84%). The monkey held down the joystick and fixated a central spot in order for a peripheral white square stimulus to appear (2 or 4 locations were tested). On most trials (90%), after a random interval (1.5–3.5 s), the peripheral stimulus dimmed, and the monkey was rewarded for releasing the joystick within 1 s after the dimming. In 10% of the trials, the peripheral stimulus did not dim, and the monkey was rewarded for continuing to hold down the joystick. The joystick task allowed us to check whether caudate neurons activities were modulated by joystick release, and by varying the location of the dimming stimulus, we could also assess preferences for visual stimulus location. A total of 57% of caudate cells show significant activity during this joystick task during at least one of these three periods (visual [0:0.5 s]; delay [0.5:1 s] or joystick release [−0.2:0.1 s]) compared to a baseline period [−0.4:0 s from stimulus onset]; most did not show any side preference ([visual: 29%/14%/57%], [delay: 23%/12%/65%], and [joystick release: 27%/21%/52%] for contralateral, ipsilateral, or neither, respectively). A small proportion of caudate neurons (8%, 18/227) were not tested with MGSs or the joystick task but only during the motion-direction change detection (CD) task.

### Motion-direction CD task

All caudate neurons were tested using a motion-direction change detection task performed covertly during maintained central fixation, essentially identical to that described previously [17], except we used a joystick rather than a button press. Briefly, the monkey started a trial by fixating a central white square and holding down a joystick for 0.25 s. A peripheral cue ring was then presented for 0.2 s to indicate which location in the visual field the animal should monitor. The cue was placed either at the neuron’s preferred location (as determined by MGSs or joystick task) or at the diagonally opposite location. We placed the cue around the horizontal meridian (<30 degrees) when no preferred location was determined by MGSs or joystick task. The location of the ring was blocked for 68 successive trials. The cue ring was extinguished, and after 0.5 s, when only the fixation point was still present, two motion patches (described below) were presented, one at the same location as the spatial cue and the other one (the foil) in the diagonally opposite location (Fig 1A). The direction of motion in the cued and foil patch was varied day to day but always differed by 90 degrees.

We placed the motion stimuli at locations expected to evoke maximal activity for each neuron, based on the modulation observed during the MGS and joystick task. The average eccentricity was 12 degrees (range: 10–13 degrees); in most cases, stimuli were placed on or near the horizontal meridian (<30 degrees). In cases for which no obvious spatial selectivity was observed during the MGS or joystick tasks, the motion stimuli were placed at an eccentricity of 12 degrees along the horizontal meridian, since these locations tended to be effective at modulating neurons in the attention task. The visual motion stimuli were circular patches of moving dots, with the direction of motion of each dot drawn from a normal distribution with a mean value (defined as the patch motion direction) and a 16-degree standard deviation. The lifetime (10 frames, 100 ms), density (25 dots/deg^2^/s), and speed of the dots (15 deg/s) were held constant. The radius of the aperture varied between 3 and 3.75 degrees, depending on the eccentricity of the patch; the median value was 3.25 degrees. Luminance of the fixation dot and of each moving dot in the motion patches was 45 cd/m^2^. The background luminance of the monitor was 9.9 cd/m^2^.

The monkey was trained to release the joystick if the motion-direction changed in the patch at the previously cued location; otherwise, he should keep holding the joystick down for trials that had no motion-direction change or a motion-direction change in the foil patch. On each trial, a single motion-direction change could occur anytime 1.0–4.3 s after the onset of the motion stimuli. The proportions of trials with a motion-direction change at the cued location or foil location or had no change were 57%, 29%, and 14%, respectively. The size of the motion-direction change was adjusted based on psychometric tests of each monkey to keep performance near threshold level (75% of performance), depending on visual field location and motion direction; the median direction changes were 28 and 26 degrees for Monkeys R and P, respectively. Clockwise and counterclockwise direction changes were equally likely and randomly chosen. After the motion-direction change, the stimuli remained on the screen for 1 s or until the animal released the joystick. Hits were defined as joystick releases that occurred within 1 s of a motion-direction change in the cued patch. False alarms were defined as incorrect joystick releases when the motion-direction change occurred at the foil location. Correct rejections were defined as successful nonreleases when no change occurred at the cued location. Monkeys were rewarded with a small drop of liquid (apple juice mixed with water) at the end of each correctly performed trial (hits and correct rejections). The monkeys were required to maintain fixation of the central square for the entire duration of the trial (until after their joystick release); otherwise, the trial was aborted.

At the beginning of each block of trials, the monkeys performed the motion-direction CD task with only one stimulus (single-patch condition) during the first 10–12 trials of each block. The single motion patch stimulus was located either at the cued location within the block (100% of the trials for Monkey R, 50% for Monkey P) or at the foil location (50% of the trials for Monkey P). For Monkey P, the presentation of the single motion patch was preceded by the presentation of the spatial cue. Monkeys were rewarded for correct detection of MC at the cued location and successful nonreleases when no change occurred at the cued location (Monkey R and P) or when a change occurred at the foil location (Monkey P only). For analysis of single-patch trials, we only used trials in which the single patch was preceded by a cue so that the sequence of stimulus events was directly comparable to the two-patch condition. Hit rates for single-patch conditions were 77.6% and 77.0% for Monkeys R and P, respectively, confirming that the size of the motion-direction change was set near the threshold level.

Data from the motion-direction CD task were collected in 105 recording sessions in Monkey R and 60 recording sessions in Monkey P. We obtained neuronal recordings (*n* = 227 neurons) for an average of 200 trials per location of spatial cue; neurons recorded for fewer than 100 trials in the task were excluded from analysis. We defined contralateral trials as trials in which the spatial cue was presented on the side of the visual field contralateral to our recording sites in the caudate nucleus. Monkeys were first trained on the task using their right hand (contralateral to the recording sites), which was used during all recordings except the 38 sessions (44 neurons) when the right and left hands were used in separate interleaved blocks.

### Neuronal peristimulus time histograms (PSTHs) and cue-related modulation

**Fig 2:**
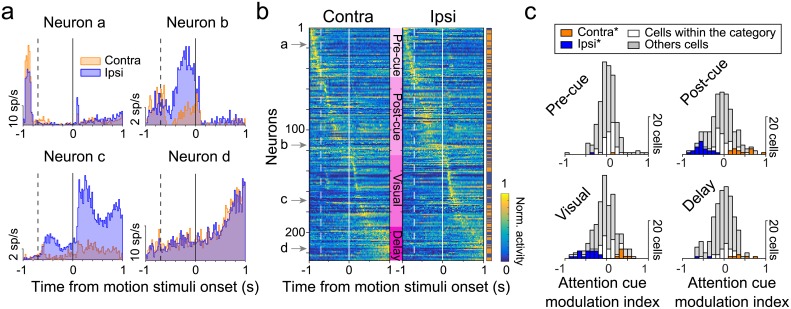
Caudate neuronal activity and cue-related modulation during the CD task. (A) Example caudate neurons. Activity of four different caudate neurons (a, b, c, and d) aligned on the onset of the motion patches (solid vertical line) when the cue was contralateral (“contra,” orange) or ipsilateral (“ipsi,” blue) with respect to the recording site. The dashed vertical lines indicate when the spatial cue was presented. (B) Normalized activity (“Norm. activity”) for our complete population of caudate neurons (*n* = 227). Each row represents the normalized activity of a single neuron for contralateral presentation of the spatial cue (left) or ipsilateral presentation (right) aligned on motion stimuli onset. We computed normalized PSTHs by dividing the raw values from each time bin by the maximum firing rate (peak of each neuron’s PSTH) across all conditions (i.e., either contralateral or ipsilateral conditions, whichever was higher). Neurons were ranked according to the time of their peak activity across both contralateral and ipsilateral cue conditions. The colored sidebar on the right indicates whether each neuron had maximal activity for contralateral (orange) or for ipsilateral (blue). Solid white lines indicate onset of motion stimuli; dashed lines indicate spatial cue onset. Neurons were grouped according to the timing of the peak activity (labels in pink): before the spatial cue onset (Pre-cue), between the spatial cue and the motion stimuli onset (Post-cue), after the motion stimuli onset (Visual), and during the delay period prior to motion-direction change (Delay). (C) Spatial cue modulation in caudate nucleus. The histograms display the distribution of attention cue modulation index values for the periods for each group of neurons. The *p*-values were corrected with the Holm’s variant of the Bonferroni method. White bars indicate the cells within the appropriate category (pre-cue, post-cue, visual, or delay), whereas gray bars illustrate the cells out of the category. Colors indicate significant side preference (orange for contralateral, blue for ipsilateral, white for neither; Wilcoxon rank sum test, *p* < 0.05). Underlying data available in S1 Data. CD, change-detection; PSTH, peristimulus time histogram; sp/s, spikes per second.

For visualizing neuronal activity, we computed PSTHs using nonoverlapping bins of 0.02 s. To visualize neuronal activity across the population of caudate neurons, we computed normalized PSTHs by dividing the raw values from each time bin by the maximum firing rate (peak of each neuron’s PSTH) across all conditions (i.e., either contralateral or ipsilateral conditions, whichever was higher). We used the timing of the peak of each neuron’s PSTH to determine the categories of neurons for Fig 2 ("pre-cue” [−0.95 to −0.7 s], “post-cue” [−0.7 to 0 s], “visual” [0 to 0.5 s], and “delay” [0.5 to 1 s] from motion stimuli onset). For analysis of neuronal activity, the firing rates within different temporal windows were computed from the trial-by-trial spike counts. To compare firing rates, we performed nonparametric statistical tests such as Wilcoxon signed rank (paired or not paired) or Kruskal-Wallis test with a significant threshold of *p* < 0.05. We corrected *p*-values using Holm’s variant of the Bonferroni method.

To quantify spatial cue–related modulation, we computed a standard attention cue modulation index defined as [R_contra_ − R_ipsi_]/[R_contra_ + R_ipsi_], where R_contra_ and R_ipsi_ are the mean activity on the contralateral trials and ipsilateral trials, respectively. Mean activity was computed in different temporal windows: “pre-cue” (−0.25 to 0.02 s) before the spatial cue onset, “post-cue” (0.2 to 0.6 s) after the spatial cue onset, “visual” (0.1 to 0.5 s) after the motion stimuli onset, and “delay” (0.5 to 1 s) after the motion stimuli onset. We compared those mean activities with baseline activity (−0.95:−0.75 s) before the spatial cue onset (Wilcoxon signed rank *p* < 0.05). This modulation index was also computed separately for trials in the single-patch condition.

### Analysis of response-choice activity

To analyze response-choice activity, we first identified neurons that showed significant changes in activity after the change in the visual motion stimulus. We aligned the data on the time of the motion-direction change and compared spike counts after the MC (0.1 to 0.6 s) to those before the MC (−0.5 to 0) and identified a subset of caudate neurons that had significantly higher activity after the MC (80/227, 35%, Wilcoxon signed rank test, *p* < 0.05). We restricted our analysis to this subset of caudate neurons. We aligned activity on the joystick release for hit responses (separately for contralateral- and ipsilateral-change trials), identified the time of peak activity by fitting a Gaussian function to the data from −0.5 to 0.5 s with respect to joystick release, and then measured the activity within a 0.3 s window centered on the peak. The spike counts from this 0.3 peak-centered window were then used for further analysis of response-choice activity.

We used a standard receiver operating characteristic (ROC) analysis [18] to determine the sensory and motor-related preferences of neurons during the response-choice epoch. For each neuron, we did three ROC-style analyses. The first analysis assessed how well response-choice activity discriminated the location of the visual MC event. We divided correctly performed trials based on where the motion-direction change occurred (hits contralateral versus hits ipsilateral); for these two different types of trials, the action was the same (releasing the joystick), but the location of the visual event was different. The area under the ROC curve (AROC) quantifies how well the location of the visual event could be discriminated based on the activity of each neuron, following a convention with values greater (less) than 0.5 indicating a preference for the contralateral (ipsilateral) side. The second analysis assessed how well response-choice activity discriminated the two behavioral outcomes (detect probability; hits versus misses); the sensory conditions were the same, but the response choice was different (release versus hold). For this analysis, outcome values greater (less) than 0.5 indicated a preference for hits (misses). The third analysis (neuronal sensitivity) assessed how well response-choice activity discriminated the presence or absence of the MC event in either motion patch (cued or foil). For this analysis, outcome values greater (less) than 0.5 indicated a preference for presence of the MC event (absence). To analyze data for trials without joystick releases, we aligned activity on the median reaction time computed for hit trials separately for contralateral and ipsilateral conditions. Significance of ROC values was evaluated using bootstrapped (1,000 iterations) 95% CIs.

We also analyzed neuronal activity for three other cases in which the joystick was released: (1) false alarms, when the monkey incorrectly released the joystick for stimulus changes at the foil location; (2) joystick breaks, when the monkey incorrectly released the joystick when neither cued nor foil stimulus changed; and (3) joystick trial end, when the monkey appropriately released the joystick at the end of correct rejection trials to initiate the next trial. Only neurons with at least five occurrences for each type of these three cases were used for analysis. Spike counts for each neuron were measured from a 0.3-s window identical to that used to analyze response choice activity as described above.

### Classification of task epochs using a linear classifier

For each trial from every caudate neuron (*n* = 227), we obtained spike counts in the motion-direction change task from 14 unique nonoverlapping epochs, defined by different time periods during the trial (*n* = 7) and whether the cue was ipsilateral or contralateral (*n* = 2). The seven time periods were (1) “pre-cue,” a 0.23-s epoch starting 0.25 s before cue onset; (2) “post-cue,” a 0.4-s epoch starting 0.1 s after cue onset; (3) “visual,” a 0.4-s epoch starting 0.1 s after motion patch onset; (4) “delay,” a 0.5-s epoch starting 0.5 s after motion patch onset; (5) “change contra,” a 0.5-s epoch starting 0.1 s after a motion-direction change in the contralateral patch; (6) “change ipsi,” a 0.5-s epoch starting 0.1 s after a motion-direction change in the ipsilateral patch; and (7) “change neither,” a 0.5-s epoch matched in time to the two preceding change epochs.

We first used the “svmtrain” and “svmclassify” functions in Matlab (version R2015b, The Mathworks, Natick, MA, US) to train and test a linear binary classifier for each of the 14 epochs defined above. For each classifier, we randomly drew (with replacement) a single-trial spike count from each neuron from the corresponding epoch to generate a single-trial feature set (*n* = 227 neurons) and then repeated this procedure multiple times (*n* = 150) to make the full data set for that classifier. Using 120/150 of the trials, each classifier was trained to distinguish data from its particular epoch from data pooled together from all of the other epochs. The remaining 30/150 trials were held in reserve to test and cross-validate the performance of the classifier with data from its own epoch. In addition, each of the 14 classifiers was also tested with the reserve data from each of the other 13 classifiers individually to generate a confusion matrix (i.e., epochs that might be identified by more than one classifier). Thus, even though each classifier was trained in a binary fashion (i.e., one epoch versus the remaining 13 lumped together), it was tested in a multiclass manner (i.e., each epoch tested individually). For each classifier, this procedure was then repeated 1,000 times, and the fifth percentile from the distribution of outcomes was compared to chance performance to identify significant results (reported as medians).

In a second analysis, we followed the same procedure using data from the epochs related to response choice (epochs 5–7 defined above, for contralateral and ipsilateral cue conditions), subdivided based on trial outcome (hit, correct reject, miss, false alarm: uncued change, and joystick breaks: no change). We trained classifiers for the four possible correct trial outcomes and then tested these four classifiers on all 10 possible trial outcomes; the training was restricted to four outcomes to avoid overfitting the data and to test whether error responses involved the same patterns of activity as correct responses. A bootstrap procedure (1,000 repeats) was used again to assess significance.

## Results

We recorded the single-unit activity from 227 caudate neurons in 2 monkeys (153 for Monkey R and 74 for Monkey P) trained to perform a motion direction CD task (Fig 1A). All related data are available in S1 Data file. The neurons were identified as PANs [10,19] based on their low background activity and were located in the head and body of the caudate nucleus within 7 mm of the AC (Fig 1C). The neuronal data were qualitatively similar across the two animals and have been pooled for simplicity.

Before describing the neuronal data, here we briefly characterize the monkeys’ performance in the attention task. As described in detail in the Materials and methods section, “hits” were defined as correct releases of the joystick when monkeys detected a change in the direction of motion at the previously cued location, and “false alarms” were defined as incorrect releases of the joystick when the motion-direction change occurred at the uncued foil location (Fig 1A). The task invoked mechanisms of spatial attention because the amplitudes of the MCs were placed near each monkey’s psychophysical threshold, and the task required ignoring irrelevant changes in the direction of motion at the uncued foil location. Both monkeys performed the task reliably across a total of 165 recording sessions (Fig 1B). The hit rates of both monkeys were 60%–64% (Monkey R: 62.2% contralateral, 62.2% ipsilateral; Monkey P: 60.4% contralateral, 64.0% ipsilateral) with only minor differences in hit rates between the two sides (R: *p* = 0.996, P: *p* = 0.070, Wilcoxon signed rank test). The false alarm rates for foil changes were low (R: 4.9% contralateral, 6.1% ipsilateral; P: 7.5% contralateral, 5.9% ipsilateral) and not different between the two sides (R: *p* = 0.196; P: *p* = 0.124, Wilcoxon signed rank test). Correct reject rates (i.e., nonreleases of the joystick when neither the cue nor foil stimulus changed) were 71%–79% (Monkey R: 71% contralateral, 74% ipsilateral; Monkey P: 79% contralateral, 79% ipsilateral). Conversely, the incorrect release rates on neither-change or foil-change trials were 21%–29% (Monkey R: 29% contralateral, 26% ipsilateral; Monkey P: 21% contralateral, 21% ipsilateral). The incorrect releases rates on cue-change trials (i.e., anticipations before the MC) were low (<5%). The two monkeys showed small (less than 20 ms) but significant differences in mean joystick reaction time for stimulus changes on the two sides (Monkey R: 0.579 s contralateral, 0.588 s ipsilateral, *p* = 0.007; Monkey P: 0.498 s contralateral, 0.516 s ipsilateral, *p* = 0.005; Wilcoxon rank sum test). Analysis of the voltage traces used to measure the position of the joystick indicated that the movements used to release the joystick were nearly identical in both monkeys across these conditions (S1A Fig). The absence of strong spatial biases in the behavior is noteworthy because our sample of caudate neurons showed patterns of activity during the performance of the attention task that often depended on whether the cue was contralateral or ipsilateral to the recording site.

### Caudate activity related to the spatial cue and motion stimulus

Caudate neurons showed several distinctive patterns of activity during the early epochs of the attention task, when the spatial cue and the motion patches were presented. To illustrate the range of activity patterns, we show the time course of spike counts (PSTHs) from four example caudate neurons, sorted by whether the spatial cue was presented contralateral (orange) or ipsilateral (blue) to the recording site (Fig 2A).

The activity of many caudate neurons was related to the location and timing of the spatial cue. Some of these neurons exhibited phasic activity that preceded the appearance of the spatial cue (Neuron a, Fig 2A), suggesting the presence of an anticipatory signal made possible by the 68-trial blocking of spatial cue conditions and the fixed temporal period (0.25 s) before the appearance of the spatial cue. The pre-cue activity of this neuron was slightly but significantly larger when the cue was contralateral than when it was ipsilateral (*p* = 0.017, Wilcoxon rank sum test, period [−0.250 to −0.020 before cue onset], 18.5 spikes per second (sp/s) on average for contralateral trials versus 14.4 sp/s for ipsilateral trials). Other neurons showed phasic activity after, and presumably evoked by, the spatial cue (Neuron b). The post-cue activity for this neuron was much larger when the cue was presented on the ipsilateral side (Wilcoxon rank sum test, period [0.1 to 0.5 s after cue onset], *p* < 0.001, 0.5 sp/s for contralateral trials versus 4.1 sp/s for ipsilateral trials).

For other caudate neurons, activity was mostly related to the presentation of the motion stimuli and the delay period of the attention task. Some neurons exhibited large phasic responses to the onset of the motion stimuli, followed by activity that extended into the delay period of the task (Neuron c, Fig 2A), with a strong preference based on the location of the spatial cue that emerged shortly after cue onset; this side preference nearly eclipsed the visual phasic response in the nonpreferred condition, even though the visual stimuli were identical across the two cue conditions. The delay period activity also varied across caudate neurons. Some showed a distinctive ramp-like pattern toward the end of the delay period without any side preference (Neuron d).

To visualize the activity patterns across our population of caudate neurons (*n* = 227) during the early phases of the task, we normalized each neuron’s spike counts and rank-ordered all of the neurons based on the times of their peak activity (see Materials and methods). The representation of these results (Fig 2B), aligned on motion stimulus onset separately for contralateral and ipsilateral cue conditions, illustrates that the sample neurons in Fig 2A were exemplars of features present across the population. Specifically, based on the timing of peak activity, we classified neurons into four different groups (indicated by labels in pink gradient). The first group of neurons (Pre-cue, light pink, *n* = 48) showed peaks of activity preceding the appearance of the spatial cue (like Neuron a). Neurons in this group tended to exhibit phasic activity for both contralateral and ipsilateral cue conditions, with slightly higher activity for contralateral (as indicated by both the higher normalized activity for the contralateral plot and the larger proportion of orange horizontal tics in the side bar of Fig 2B).

The second group of neurons (Post-cue, *n* = 76) increased their activity after the presentation of the spatial cue (like Neuron b). These post-cue neurons tended to show higher activity in the ipsilateral cue condition (as indicated by the higher normalized activity and the larger proportion of blue tics in the side bar of Fig 2B). The timing of this activity was distributed across the post-cue epoch, including just after cue onset (neurons #50–60), after cue offset (neurons #80–100), and just before motion stimuli onset (neurons #115–120).

The third group of neurons (Visual, *n* = 70) had responses that appeared to be evoked by the onset of the motion stimuli (like example Neuron c). These visual neurons sometimes also exhibited cue-related activity before motion stimuli onset and lower sustained activity into the delay period. The phasic visual response showed a preference for the ipsilateral cue condition (neurons #140–170), whereas the preferences during the delay period were more equally split.

For the last group of neurons (Delay, *n* = 33), the peak of activity occurred well after the presentation of the motion stimuli and into the delay period (0.5 s and longer after motion stimuli onset). These “delay” neurons tended to show a ramping pattern of activity (like example Neuron d), similar to that described previously [5], and had a slight preference for the contralateral cue condition. The categorization of caudate neurons did not depend on the particular procedure used to rank the neurons based on the timing of their peak activity (S2 Fig).

### Modulation of caudate neurons by contralateral and ipsilateral cues

To quantify the cue-related modulation, we computed a modulation index for spike counts within different temporal periods (Fig 2C, see Materials and methods) and found clear distinctions between the groups of caudate neurons. The neurons with prominent pre-cue activity (*n* = 48) were weakly modulated by the spatial cue condition (Fig 2C). Only 4% (2/48) of this group showed significantly different activity based on cue condition, one preferring contralateral and the other one preferring ipsilateral). In contrast, the post-cue and visual neurons were strongly modulated by spatial cues. Most of the post-cue neurons (34/76, 45%) displayed a significant effect of cue condition, with almost 2/3 showing a preference for ipsilateral (22/34, 64%). Similarly, some visual neurons (29/70, 41%) showed significant cueing effects, again mostly in favor of the ipsilateral cue condition (21/29, 72%). The amplitude of the cueing effects for post-cue and visual neurons was large—the median cue modulation index was −0.55 and −0.46 (post-cue and visual, respectively) for the ipsilateral condition and 0.35 and 0.34 for the contralateral condition. We observed the ipsilateral bias for post-cue and visual periods also through the full population of caudate neurons (*t* test, *p* < 0.05) while the distributions of Attention Modulation Indices (AMIs) for pre-cue and delay periods were centered on 0 (*t* test, *p* > 0.05). Finally, neurons defined by their delay period activity were also modulated by spatial cues; a quarter of the neurons (8/33) showed a significant effect, with a slight preference for the contralateral side (6/8).

### Dependence of spatial selectivity on the visual configuration

We unexpectedly found that the spatial cue modulation was dependent on the presence of a visual distracter during the covert attention task. For visual neurons (*n* = 70), we compared activity during the standard two-patch version of the attention task with activity during a simpler single-patch version that omitted the distracter. During this single-patch task, a single motion patch was presented at the contralateral or ipsilateral location, and the animal was again rewarded for releasing the joystick if the motion direction in the single patch at the cued location changed.

**Fig 3:**
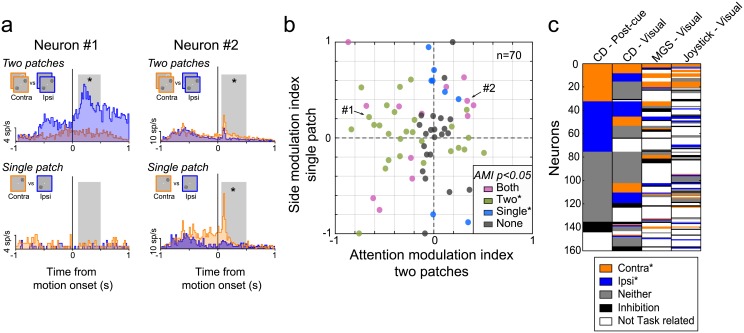
Dependence of caudate visual activity on the presence of distracters. (A) Firing rates for two sample caudate neurons aligned on presentation of visual stimuli for the two-patch condition (top row) and single-patch condition (bottom row). The orange/blue code indicates respectively the spatial cue location or the single patch location for contralateral (“contra”)/ipsilateral (“ipsi”) side. Gray boxes demarcate the 0.4-s time period used for computing the AMI for the two-patch condition and SMI for the single-patch condition. Asterisks indicate significant values for AMI/SMI (Wilcoxon rank, *p* < 0.05). (B) Scatter plot of AMI and SMI values computed for each caudate neuron with visual activity (*n* = 70). AMI values on the x-axis greater (less) than 0 indicate preference for contralateral (ipsilateral) hits in the two-patch condition. SMI values on the y-axis greater (less) than 0 indicate preference for contralateral (ipsilateral) for single-patch condition. Each dot represents one caudate neuron from the "visual" subpopulation (Fig 2B, *n* = 70). Color indicates the neuron’s group assignment based on the AMI/SMI values: AMI for two patches different for one side (green), both different from chance (purple), SMI for single patch different for one side (blue), and neither different from chance (gray). (C) Side preference for the motion CD task, MGS task, and joystick task in the population of 160 caudate neurons tested with the three tasks. Each row represents a single neuron across different visual epochs: post-cue and visual for CD task and visual periods for MGS and joystick mapping task. Neurons were sorted according their side preference for post-cue period. Colors indicate side preference (orange for contra, blue for ipsi, gray for neither) when the activity was significantly greater than the baseline (Wilcoxon rank sum test, *p* < 0.05), when no significant activity was reported (Wilcoxon rank sum test, *p* ≥ 0.05; white), or when activity was significantly lower than the baseline (Wilcoxon rank sum test, *p* < 0.05; black). We used all trials (hits, misses, false alarms, and correct rejects) for the CD trials and all correctly performed trials for the MGS and joystick tasks. Underlying data available in S1 Data. AMI, Attention Modulation Index; CD, change-detection; MGS, memory-guided saccade; SMI, Side Modulation Index; sp/s, spikes per second.

This comparison revealed that the visual activity of many caudate responses was selective for visual conditions that included the second distracter patch. For example, Neuron #1 in Fig 3A showed a strong preference during the two-patch attention task for ipsilateral placement of the cued stimulus (blue versus orange, top left quadrant). This preference completely disappeared during the single-patch condition (lower left quadrant), demonstrating that the spatial selectivity of this neuron was specific to visual conditions in which an ipsilateral cued stimulus was accompanied by a contralateral distracter. Some caudate neurons were like Neuron #1 and displayed spatial selectivity during the two-patch version of the covert attention task but lost their side preference during the single-patch condition (green dots in Fig 3B, *n* = 27/70, significant AMI for two-patch but not single-patch condition); a majority of these neurons (20/27) had a preference for the ipsilateral side (i.e., AMI < 0 for the two-patch condition).

Less common were neurons like Neuron #2 (Fig 3A), which exhibited a side preference during both single-patch and two-patch conditions (violet dots, *n* = 13). Most of these neurons (9/13) retained a consistent side preference across visual conditions like Neuron #2, whereas 4 neurons preferred the ipsilateral side in the two-patch condition but preferred the contralateral side in the single-patch condition. An additional eight neurons had a significant side preference for the single-patch but not the two-patch condition (blue dots, *n* = 8). Finally, some neurons (gray dots, *n* = 22/70) did not show a side preference during either the single-patch or two-patch condition.

The visual activity of some caudate neurons was selective to the visual configuration during the covert CD task and could not be predicted by either the MGS or joystick mapping tasks. In a population of 160 neurons tested in all three tasks, we found that most caudate neurons did not show the same spatial selectivity (Fig 3C) across the different tasks. For the neurons that preferred the contralateral side during the post-cue period of the CD task (left column, orange rows, *n* = 32/160), only 10/32 showed the same spatial selectivity for MGS and joystick task during the visual periods, and only 2/43 neurons showed congruent selectivity for the ipsilateral side.

In summary, we observed cue-related modulation across all of the early epochs of the attention task, indicating that these cueing effects were not related to the delivery of the reward or to behavioral outcomes at the end of the trial. The effects were strongest in the post-cue and visual epochs and larger for ipsilateral than contralateral spatial cues. The visual cue–related modulation for many caudate neurons depended on the visual configuration—it was specific to the CD paradigm, required the presence of a visual distracter, and disappeared when only a single visual stimulus was presented at the preferred location.

### Activity of caudate neurons related to response choice

As might be expected from previous results implicating the caudate nucleus in movement sequencing and procedural learning, a subset of our caudate neurons was modulated during the joystick response choice. However, even among these neurons, neuronal activity was not simply movement related but also exhibited unexpected selectivity for the visual and task conditions.

**Fig 4:**
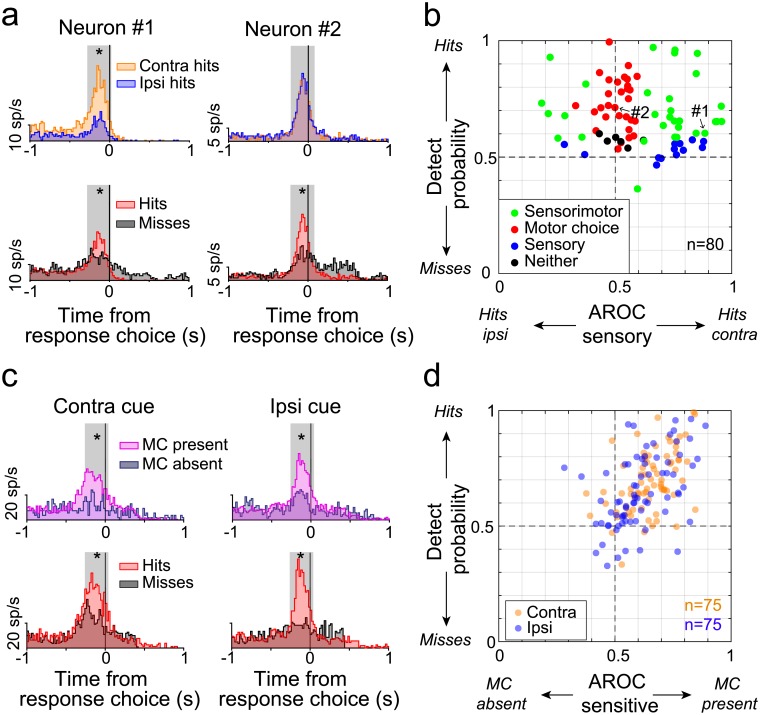
Response-choice activity in caudate nucleus. (A) Firing rates for two sample caudate neurons (#1 and #2) aligned on the joystick release. The upper row shows the response-choice activity for contralateral hits (“contra,” orange) and ipsilateral hits (“ipsi,” blue); the lower row shows activity for hits (red) and misses (gray) pooled across stimulus locations. Gray boxes demarcate the 0.3 s time period used for the ROC analysis. A top asterisk indicates that the AROC value was significantly different from chance level (0.5). For miss trials, which did not contain joystick releases, data for each neuron were aligned on the median reaction time during the recording session. The median reaction times were 0.56 s (IQR = 0.18, contralateral trials) and 0.53 s (IQR = 0.14, ipsilateral trials) for neuron #1 and 0.55 s (IQR = 0.13) and 0.52 s (IQR = 0.13) for neuron #2. (B) Scatter plot of detect probabilities and sensory ROC values computed for each caudate neuron that showed response-choice activity. AROC values on the x-axis greater (less) than 0.5 indicate preference for contralateral (ipsilateral) hits. Each dot represents one caudate neuron (*n* = 80). Color indicates the neuron’s group assignment based on the AROC values: hits versus misses AROC different from chance (“motor choice,” red), contralateral hits versus ipsilateral hits different from chance (“sensory,” blue), both different from chance (“sensorimotor,” green), and neither different from chance (black). (C) Firing rate for one caudate example aligned on the joystick release. The upper row shows the response-choice activity in the presence (pink) or absence (blue) of the MC; the lower row shows activity for hits (red) and misses (black). For the MC-present trials (pink), the change event could happen at either location (cued or foil). The left column shows responses for the contralateral trials; the right, for the ipsilateral trials. Gray boxes demarcate the 0.3-s time period used for the ROC analysis. The top asterisk indicates that the AROC value is significantly different from chance level (0.5). For miss trials and some MC-absent trials, which did not contain joystick releases, data for each neuron were aligned on the median reaction time during the recording session. The median reaction times were 0.48 s (IQR = 0.14, contralateral trials) and 0.50 s (IQR = 0.12, ipsilateral trials). (D) Scatter plot of the detect probabilities as a function of the neural sensitivity (AROC "sensitive") for the caudate neurons with response-choice activity. Only neurons with at least five occurrences for each type of conditions were used for analysis (*n* = 75/80). The color code indicates the location of the spatial cue; contralateral (orange) and ipsilateral (blue). AROC values on the x-axis greater (less) than 0.5 indicate a preference for presence (absence) of the MC; AROC values on the y-axis greater (less) than 0.5 indicate a preference for hits (misses). Underlying data available in S1 Data. AROC, area under the receiver operating characteristic curve; MC, motion change; ROC, receiver operating characteristic; sp/s, spikes per second.

Among the subset of neurons with activity modulated during the response choice (*n* = 80, defined in Materials and methods), we found that response-choice activity could be driven by sensory signals (i.e., where the MC happened), motor-related signals (i.e., whether or not the animal released the joystick), or combinations of the two. For example, the response-choice activity of Neuron #1 in Fig 4A combined a preference for contralateral over ipsilateral motion-direction changes (top) with a preference for hits over misses (bottom). To quantify these sensory and choice-related signals, we used ROC analyses to measure (1) the neuronal MC selectivity by comparing spike counts on contralateral hits to ipsilateral hits (AROC sensory) and (2) the detect probability by comparing spike counts on trials with hits versus misses (AROC motor). For Neuron #1, both ROC analyses were significantly greater than chance (AROC of 0.88 and 0.6, sensory and motor, respectively), confirming that the response-choice activity of this neuron combined a preference for hits with a preference for stimulus changes in the contralateral visual field. In contrast, Neuron #2 preferred hits over misses (AROC: 0.71) but had no preference between contralateral and ipsilateral change-event locations (AROC: 0.5), suggesting that the response-choice activity of this neuron was predominantly related to the joystick release. Similar mixed dependencies were observed across the caudate neurons with response-choice activity. Some caudate neurons were like Neuron #1 and combined a preference for change-event location with a preference for hits over misses (green dots in Fig 4B, *n* = 32/80, both AROCs were significantly different from chance level 0.5); most (23/32) of these neurons preferred contralateral change events (AROC > 0.5), with an almost exclusive preference for hits (31/32). Almost as common were neurons like Neuron #2, which signaled the motor choice without a preference for change-event location (red dots, *n* = 30). Finally, a smaller number of neurons (blue dots, *n* = 13) exhibited response-choice activity that discriminated the location of the MC (and generally preferred contralateral) but did not show any difference between hits and misses. The remaining six neurons showed selectivity for neither (black dots). Despite these distinctions, caudate neurons did not form exclusive categories based on their response-choice activity but showed a continuum of mixed preferences for change-event location and motor choice, as illustrated by the broad scatter of data points in Fig 4B. We also confirmed that aligning the response-choice activity on the time of the motion-direction change rather than on the time of joystick release (S3 Fig) did not change the proportions of caudate neurons in these different categories (sensorimotor, motor, sensory, and neither).

We also tested the time course of AROC sensory and the detect probabilities for the entire duration of the trial (see S4 Fig). Consistent with Fig 2, spatial selectivity was evident after cue onset and motion stimulus onset but also after the MC (S4 Fig). Significant detect probabilities were not present early in the trial (i.e., after motion stimulus onset) but increased markedly after the motion-direction change (S4C Fig), suggesting that the commitment to release the joystick was triggered by the motion-direction change event itself rather than formed endogenously earlier in the trial.

We next tested the modulation of caudate responses to the presence or absence of the change event. Fig 4C shows an example of a typical caudate neuron whose activity was significantly modulated by the presence or absence of the MC and also for hits versus misses, independently of the cue location (contralateral/ipsilateral). We measured the correlation between neuronal sensitivity and detect probability across our population of neurons, separately for contralateral and ipsilateral cue conditions (see Materials and methods). Neuronal MC sensitivity was significantly and positively correlated with the detect probability for both contralateral and ipsilateral cueing conditions (Fig 4D, contralateral trials, *R* = 0.49, *p* < 10^−6^; ipsilateral trials, *R* = 0.49, *p* < 10^−6^), indicating that caudate neurons with greater sensitivity to the visual event were also more strongly predictive of the response choice. Among these response-choice neurons, there was no evident preference for a particular epoch earlier in the trial (S1 Table).

Together, these results show that during the response choice itself, the activity of many caudate neurons was selective not only for the motor choice but also for the spatial location of the relevant stimulus event. Moreover, caudate neurons that better detected the presence or absence of the MC were also better at predicting whether or not the monkey would release the joystick. These findings are consistent with caudate neurons establishing a link between the occurrence of a specific sensory event and the decision to commit to a particular motor response.

### Dependence of response-choice activity on attention task conditions

The phasic response-choice activity was also dependent on the task condition in which the joystick was released. We considered three other situations during the covert attention task when the animal released the joystick, in addition to the case of “hits” analyzed in Fig 4. First, we analyzed activity during “false alarms,” when the animals incorrectly released the joystick for a MC that occurred at the foil location. Second, we analyzed “joystick breaks,” when the animals released the joystick but there was no MC event at either stimulus location. Third, we analyzed activity during “joystick releases” at the end of correct reject trials, when the animal was obliged to release the joystick in order to end the trial. We verified that the dynamics of the joystick release was the same across different trial outcomes by analyzing the voltage changes associated with the joystick movement (S1 Fig).

**Fig 5:**
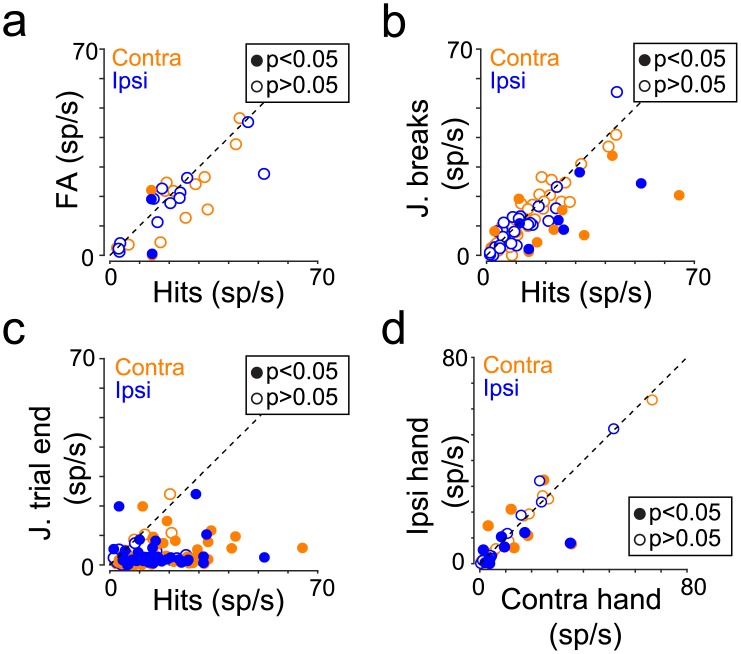
Error trials and hand influence. (A, B, C) Scatter plots of mean caudate activity during FAs (A), joystick breaks (“J. breaks”) (B), and joystick trial end (“J. trial end”) (C) as a function of mean activity to hits for contralateral trials (“Contra”, orange) and ipsilateral trials (“Ipsi”, blue). Filled dots (orange or blue) indicate when mean activities were significantly different (Wilcoxon rank sum test, *p* < 0.05, corrected with the Holm’s variant of the Bonferroni method). Dashed lines represent identity lines. Joystick breaks are defined as trials when the animals released the joystick but there was no MC event at either stimulus location and joystick trial end as joystick releases at the end of correct reject trials, when the animal was obliged to release the joystick in order to end the trial. We performed these analyses on response-choice neurons for which we recorded at least five trials for each condition (FA, joystick breaks, or joystick trial end). (D) Scatter plot of mean responses for contralateral and ipsilateral hits (orange and blue) computed during the 0.3-s time period when animals used either their ipsilateral hand (y-axis) or contralateral hand (x-axis) with the joystick. Same convention as A, B, and C. Underlying data available in S1 Data. FA, false alarm; MC, motion change; sp/s, spikes per second.

Mean activity for false alarms was not different from that for hits for most of the neurons (Fig 5A; 12/14, 86% contralateral; 12/14, 86% for ipsilateral, Wilcoxon rank sum test, *p* > 0.05). Similarly, mean activity for joystick breaks was equivalent to activity for hits for most of the neurons (Fig 5B; 35/46, 76% contralateral; 25/31, 81% for ipsilateral, Wilcoxon rank sum test, *p* > 0.05). In contrast, mean activity for hits was significantly larger than mean activity for joystick release at the end of correct-reject trials (Fig 5C, Wilcoxon signed rank, *p* < 0.001 for contralateral, *p* < 0.001 for ipsilateral). These results indicate that the response-choice activity was specific to joystick releases associated with the choice about the visual motion stimulus.

As an additional test of the specificity of this response-choice activity, for a subset of caudate neurons, we tested whether the hand used to release the joystick made a difference. We defined the contralateral and ipsilateral hand relative to the recording site of the neurons, as we did for spatial cue location. We recorded a total of 44 neurons in 38 behavioral sessions (*n* = 25 for Monkey R and *n* = 13 for Monkey P). Behavioral performance was not different when animals used their contralateral hand or the ipsilateral hand with only minor differences in hit rates between the two hands (R: 65.2% ipsilateral hand, 66.3% contralateral hand, *p* = 0.777, Wilcoxon signed rank test; P: 57.7% ipsilateral hand, 65.4% contralateral hand, *p* = 0.002). Among the population of neurons that showed choice-related phasic activity (*n* = 20), there was no preference for one particular hand. Indeed, mean responses for correct responses to MCs on the contralateral side (orange dots) or ipsilateral side (blue dots) did not depend on which hand was used to release the joystick (Wilcoxon rank, *p* = 0.681 contralateral change, *p* = 0.575 ipsilateral change, Fig 5D). Thus, the features of the phasic activity related to joystick release support the use of the term “response-choice”—this activity was specific to joystick releases associated with choices about the visual motion stimulus but was largely unaffected by which hand was used.

**Fig 6:**
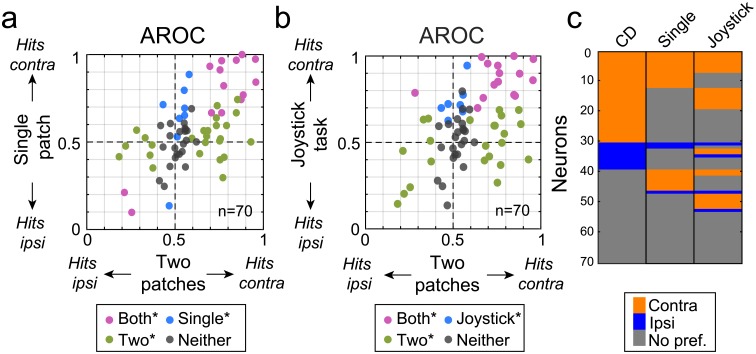
Influence of task context on response-choice activity. (A) Scatter plot for each caudate neuron (*n* = 70) with response-choice activity comparing AROC values computed for single-patch and two-patch conditions. AROC values on the x-axis greater (less) than 0.5 indicate preference for contralateral (“contra”) (ipsilateral [“ipsi”]) hits for two-patch condition; AROC values on the y-axis greater (less) than 0.5 indicate preference for contralateral (ipsilateral) hits for single-patch condition. Color indicates the neuron’s group assignment based on the AROC values: contralateral hits versus ipsilateral hits different from chance for two patches only (Two*; green), contralateral hits versus ipsilateral hits different from chance for single patch only (Single*; blue), contralateral hits versus ipsilateral hits different from chance for both conditions (Both*; purple), and neither different from chance (Neither; gray). (B) Scatter plot of AROC values computed for each caudate neuron tested with the dimming joystick task (*n* = 70) with response-choice activity for two-patch and dimming joystick conditions. AROC values on the y-axis greater (less) than 0.5 indicate preference for contralateral (ipsilateral) hits for dimming joystick condition. Same conventions as A except that blue color indicates when responses to contralateral hits versus ipsilateral hits are different from chance for joystick task only (Joystick*; blue). (C) Side preference for the motion CD task for CD with two patches (CD), single patch, and joystick task in the population of 70 caudate neurons tested with these three conditions. Each row represents a single neuron across different task conditions during the response choice period (0.3 s): two patches (CD), single patch, and joystick mapping task. Neurons were sorted according their side preference for CD task. Colors indicate side preference (orange for contralateral, blue for ipsilateral, gray for neither [“No pref.”]). Underlying data available in S1 Data. AROC, area under the receiver operating characteristic curve; CD, change detection.

However, the response-choice activity was strongly affected by the visual and task conditions (Fig 6). We compared caudate neuronal activity for joystick releases during three different conditions: (1) the standard two-patch attention task, (2) the single-patch version of the attention task introduced earlier, and (3) a joystick task in which the monkey released the joystick when a single peripheral square stimulus reduced its luminance. Many caudate neurons exhibited response-choice activity only during the two-patch condition (green dots in Fig 6A, *n* = 25/70, AROC different from chance level 0.5 for two patches only) and did not show any side preference during the joystick task (green dots in Fig 6B, *n* = 21/70). Another group of caudate neurons showed a dependence on the location of the visual stimulus evoking the joystick release, and this side preference was retained across the three different task conditions (Fig 6A, violet dots, *n* = 14/80) and also during the dimming joystick task (Fig 6B, violet dots, *n* = 21/70). For all of these neurons, the side preference remained the same across visual conditions (Fig 6A, *n* = 14/14) and tasks (Fig 6B, *n* = 19/21).

To facilitate comparison of neuronal activity across these three task conditions (two patches, single patch, or joystick task), we illustrated the spatial selectivity of each neuron (*n* = 70) using a color-coded format similar to that used in Fig 3C. Some caudate neurons (15/39, 38%) lost the spatial selectivity exhibited during the attention task when we tested them with the other tasks (Fig 6C, rows colored blue or orange for “CD” but gray for “single patch” and “joystick task”), indicating that the spatial selectivity was dependent on the presence of the distracter stimulus even during the response-choice period. However, in contrast to the side preference exhibited during earlier trial epochs (e.g., Fig 3c), most neurons with a side preference during the response choice preferred the contralateral side during the two-patches version (left column, orange rows, *n* = 30/70).

Overall, like the visual cue–related modulation of caudate neurons described earlier, the response-choice activity was also often specific to the visual and task conditions—in particular, the presence of a visual distracter—even though the act of releasing the joystick was the same and was largely unaffected by which hand was used.

### Classification of epochs of the attention task based on caudate activity

**Fig 7:**
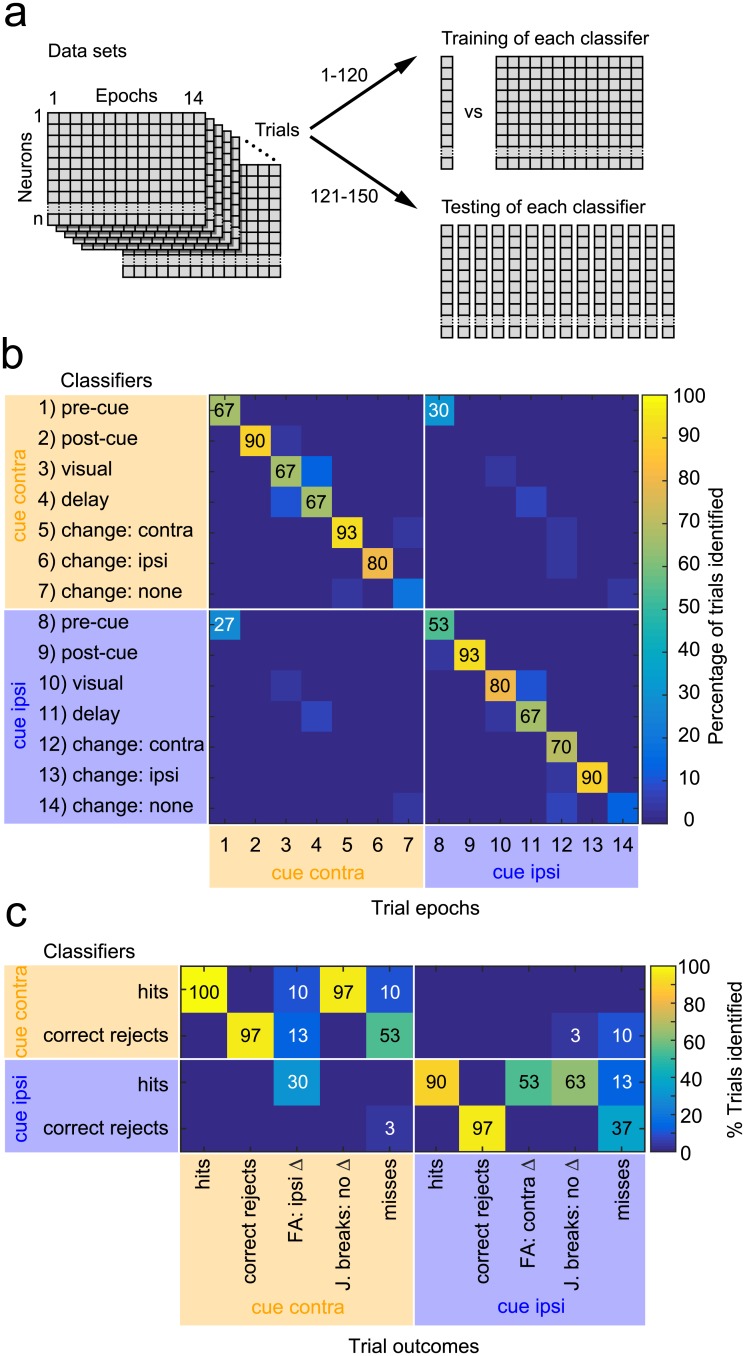
Linear classifier performance for the covert attention task. (A) Cartoon illustrating how the linear binary classifiers were trained and tested. Each single-trial data set was generated by random draws of spike counts from each neuron for each of the 14 epochs. For training of each classifier, trials 1–120 of these data sets were used to construct a classifier that could distinguish between data from its own epoch and data pooled across the other 13 epochs. For testing of each classifier, trials 121–150 were used to test and cross-validate the classifier with data from its own epoch individually and also to test how frequently it incorrectly recognized each of the other 13 epochs individually. This procedure was repeated 1,000 times to obtain the median values shown graphically in the other plots. (B) Linear classifiers applied to caudate activity from different epochs of the attention task. The matrix illustrates the percentage of trials positively identified, as indicated by the color scale, by each of the 14 classifiers (rows) for each of the 14 trial epochs (columns). The diagonal of the matrix corresponds to the percentage of trial epochs correctly identified by each classifier; values outside the diagonal indicate the percentages of trial epochs that were incorrectly identified by classifiers trained on other epochs. Numbers overlaid on the matrix indicate percentage scores for every case that was significantly greater than chance performance, for correct identifications (black numbers along diagonal) and for incorrect identifications (white numbers). (C) Linear classifier performance for trial outcomes during the covert attention task. The matrix illustrates the percentage of trials positively identified, as indicated by the color scale, by each of the four classifiers trained on change-epoch data from correct trials (rows) for each of the 10 possible trial outcomes (columns). Numbers overlaid on the matrix indicate percentage scores for every case that was nonzero, for values greater than chance performance (black numbers), and for values less than chance performance (white numbers). FA, false alarm; J. break, joystick break.

Our previous analyses illustrated that caudate neurons exhibited diverse patterns of activity that appeared to cover the different trial conditions and the full time course of the attention task, including the visual periods and also the response choice. We next wanted to confirm this result by testing whether there was sufficient information contained in the spike counts from our population of caudate neurons to correctly identify the epochs and cue conditions in the attention task using linear classifiers (support vector machine [SVM]). As illustrated in Fig 7A and described in more detail in the Materials and methods section, we identified 14 unique epochs based on the cueing condition and trial events in the attention task, trained a separate classifier for each of these epochs, and then cross-validated and quantified the performance of each classifier by testing it with spike counts from its own epoch as well as each of the other 13 epochs individually. To visualize the results, in Fig 7B, we display the performance of each classifier in matrix format, using a color scale to indicate the percentage of test trials identified by each classifier for each of the 14 trial epochs.

The set of classifiers performed significantly above chance for 12 of the 14 epochs, with no significant misclassifications except for the pre-cue epochs (Fig 7B). The post-cue epochs and MC epochs were classified with higher accuracy (90% and 93% correct), regardless of whether the cue was contralateral or ipsilateral. The pre-cue epochs were also classified better than chance (67% and 53% correct), presumably reflecting the fact that cue conditions were blocked, although the two pre-cue epochs were also the only cases with significant, mutual misclassifications (30% and 27% errors), maybe because these epochs are less valuable for representing the task sequence compared to the other ones. The two no-change epochs (“change: none” in Fig 7B) were the only epochs that were not correctly identified above chance level.

### Classification of trial outcomes during the attention task

Because caudate neuronal activity was related to response choice and also contained sufficient information to identify epochs of the attention task (Fig 7B), we tested whether the spike counts from our population of caudate neurons could identify the trial outcomes during the attention task. Specifically, we sought to determine if caudate activity during the MC epoch was related to the stimulus condition or to the perceptual choice (Fig 7C). To this end, we subdivided the data from the change epoch used previously for the linear classifier analysis (epoch 5–7 and 12–14, Fig 7B) based on the trial outcome (hit, correct reject, miss, false alarm, and joystick break). Consistent with the previous analysis, we subdivided erroneous releases of joystick into two types—joystick releases for MC on the uncued side (false alarms) and joystick releases for no MC (joystick breaks). We adopted the strategy of training classifiers on data from the four types of correct trials (hits and correct rejects for contralateral and ipsilateral cue conditions) and then testing these four classifiers with data from all 10 trial outcomes.

The results demonstrate that caudate neuronal activity represents the response choice, correct or incorrect, especially when the cue is on the contralateral side (Fig 7C). Classifiers identified correctly performed trials (hits and correct rejects) with high probability (90%–100%); these same classifiers also identified particular classes of errors. For example, the classifier for contralateral hits also identified 97% of joystick breaks committed during contralateral cues with no MC (upper-left row of matrix in Fig 7C), consistent with erroneous detection of an MC that did not happen. On the other hand, the contralateral hits classifier only rarely identified false alarms committed during contralateral cues with ipsilateral MC (10%); these were more frequently identified by the ipsilateral hits classifier (30%), consistent with erroneous coding of the cue condition. The classifier for contralateral correct rejects also identified 53% of misses committed during contralateral cues, suggesting that the motion-direction change was simply not detected. For the ipsilateral classifiers, the outcomes were slightly different. The classifier for ipsilateral correct rejects also identified misses (37%), but the classifier for ipsilateral hits identified both types of erroneous releases (false alarms and joystick breaks) committed during ipsilateral cues (53% and 63%), albeit with lower probabilities.

These results quantitatively demonstrate that the pattern of spike counts across our caudate neurons represented the perceptual choices made during the covert attention task, on both correct and error trials. Choices during contralateral and ipsilateral cue conditions are both represented, but the patterns associated with erroneous releases (false alarms and joystick breaks) are different between the two cue conditions.

## Discussion

By recording neuronal activity in the caudate nucleus during a covert visual attention task, our results provide new insight into the role of the caudate in selection and covert attention processes. Because our task involved a nonspatial response (holding or releasing a joystick) guided by stimuli presented in the right or left visual field, we were able to document the visual-cue selectivity of caudate neurons during the covert attention task, dissociated from selectivity for the spatial goal of the movement. We also found that there was sufficient information in the pattern of activity across our population of caudate neurons to correctly identify the different epochs during the visual attention task, as well as the response choice, regardless of cueing condition. These results are consistent with the view that the basal ganglia are important for representing the sequence of belief states that underlie action selection [7], and illustrate how this type of mechanism might apply to perceptual and cognitive functions even when no overt action is involved.

### Spatial selectivity of caudate neurons during covert attention task

We found that the activity of caudate neurons was strongly modulated by spatial cues during the covert attention task, consistent with previous findings that neurons in the caudate head are modulated by task instructions [10,19]. Indeed, the size of the cue-related modulation we found for caudate neurons was much larger than what has been typically reported for visual cortical areas during visual attention tasks similar to ours. The firing rates of our caudate neurons were modulated by spatial cues by more than 100% (median modulation indices were 0.35–0.55, Fig 2C), compared with the more modest changes of approximately 8% found for visual area V1 [20], 10%–20% for middle temporal area (MT) [21], approximately 26% for visual area V4 [20], approximately 40% for medial superior temporal area (MST) [22,23], and 25%–50% found for lateral intraparietal area (LIP) [24,25]. The attention cue–related changes we observed for caudate neurons are more consistent with the larger cueing effects found for neurons in frontal cortical areas such as the frontal eye fields [26] and dorsolateral prefrontal cortex [27]. Our caudate recordings also included a substantial number of neurons that showed higher activity for ipsilateral spatial cues, similar to what has been found for dorsolateral prefrontal cortex [27] and unlike the preference for contralateral spatial cues found in visual cortex. These similarities in spatial cueing effects between frontal cortex and our caudate neurons are consistent with the known anatomy. The frontal cortex provides direct projections to regions in the head of the caudate nucleus that overlap with the locations of our recording sites [28]; visual cortical areas implicated in our CD task would be expected to project to more posterior regions around the genu of the caudate nucleus [29].

The large and bilateral spatial cueing effects we found for caudate neurons are presumably related to aspects of selective attention that lie outside of the changes in sensory processing. One possibility is that these cueing effects are related to spatial working memory. Selective attention and working memory are often studied as separate behavioral phenomena, but performing an attention task requires remembering the location of the cue, and conversely, there is substantial evidence that working memory may rely on rehearsal using visual selection mechanisms [30,31]. Neurons in dorsolateral prefrontal cortex are well known for the sustained delay-period activity that could support working memory [32,33], but this sustained activity is also consistent with representing the currently attended location [34]. Our results demonstrate that similar signals are present on caudate neurons, and the design of our selective attention task allows us to attribute this activity to visual working memory rather than to movement planning, because our task involved a nonspatial joystick response and no targeting saccades. This interpretation is consistent with recent caudal evidence that the output of the basal ganglia biases sensorimotor processing that takes place in other structures but is not necessarily the route responsible for evaluating the sensory evidence itself [6,35].

Another possibility is that the spatial selectivity was related to reward expectation, which is often closely linked to spatial attention [36]. For example, previous studies showed that visual or memory-related responses of caudate neurons may be modulated by the expectation of reward [37]. Indeed, caudate neurons can exhibit selective activity for visual cues that predict the direction of rewarded saccadic eye movements when those visual cues are defined by spatial location [38,39] or by nonspatial features such as color [37]. In our task, the delivery of reward was not related to a goal-directed movement, because animals responded by releasing or holding down the same joystick across all task conditions. We also showed that spatial selectivity cannot be explained by the expectation of reward in general, because it was present for the covert attention task but not for other task conditions (single patch, joystick task, and MGS task). Thus, although it is difficult to completely rule out some relationship with reward expectation, our results would point to a novel form that is specifically associated with the conditions of our covert spatial attention task.

We also showed that the cue-related modulation of caudate neurons was strongly dependent on the visual and task conditions. One possible explanation is that this selectivity is a byproduct of movements that are planned but then not executed: subjects in our covert attention task might have preferred to look directly at the cued motion patch if they had been allowed to break central fixation. However, the properties of our caudate neurons are not consistent with this explanation. We found that the spatial selectivity exhibited during the attention task was not well predicted by the spatial preferences during MGSs or the other joystick mapping task, indicating that it is unlikely that the cue-related modulation during the attention task was simply due to movement planning. However, it remains possible that some of the cue-related modulation was related to subtle movements of the body that might also depend on the visual and task conditions.

In addition, the visual-evoked activity of caudate neurons was specifically modulated during the covert attention task when the second distracter patch was present but not when only a single patch was presented. Similarly, the response-choice activity of many caudate neurons was specific to motion-direction changes in one visual hemifield only when a distracter was present, even though the physical act of releasing the joystick was the same across all tasks and did not involve a goal-directed movement. These results demonstrate that choice-related activity in the caudate includes the covert spatial selection of behaviorally relevant events in addition to the overt preparation of orienting movements [5,40].

We also found that in caudate nucleus, sensitivity and detect probability were correlated, suggesting that the perceptual decision might be based on the activity of the most sensitive neurons; similar results have been shown in cortical areas MT [41] and LIP [42]. These observations are consistent with the caudate being part of the circuit for implementing the perceptual decision during the covert attention task.

### Caudate activity and task sequencing

We found that different subsets of caudate neurons showed phasic cue-related modulation during particular time epochs so that the population of neurons tiled the full duration of the task, even during the response choice. Previous studies have demonstrated that caudate neurons can represent a task sequence when animals perform a sequence of oriented movements [4,14] and that caudate activity tiles the full duration of the trials [43]. Because our covert attention task did not include overt movements to the attended location, our results are substantively different from these previous findings and thus demonstrate activity related to task sequence rather than action sequence. This type of sequential transient activation of caudate neurons is strikingly similar to a pattern recently reported in the dorsomedial striatum of rats during a non-match to position task [15]; as in our results, this sequential activity could not be explained by changes in motor activity and was present over the entire task, not just the delay period. One might argue that the sequential activation of caudate neurons was simply related to time and not the task sequence itself. However, the cue-related modulation provides compelling evidence that activity depended on the sequence of events—for example, many caudate neurons showed visual- and choice-related activity that was specific to the visual field location of the preceding spatial cue even though the timing of events was the same across these conditions.

Our results are also consistent with the idea that the striatum plays a central role in representing the “belief states” needed to guide action selection [7]. Using a set of linear classifiers, we found that the pattern of activity across our population of caudate neurons correctly identified trial epochs throughout the visual attention task. Because most of these trial epochs involved maintained fixation and the absence of any overt action, these results demonstrate that caudate activity is not only important for tracking sequences of actions, as previously shown [4,14], but also for tracking the sequence of seemingly quiescent behavioral states that are necessary to perform a covert attention task [44].

This perspective nicely dovetails with recent evidence showing that caudate neuronal activity encodes several aspects of perceptual decision-making during a visual motion direction–discrimination task [5]. As in our data set, the caudate neurons recorded by Ding and Gold [5] showed a strong dependence on sensory and task conditions, a diversity of activity patterns and timing, and that only a subset of neurons had activity linked to the action—in their case, a saccade to one of two choice targets. They interpret their results within the framework of accumulation of sensory evidence toward a decision boundary [45], which has been an enormously fruitful approach; it may be useful now to consider how this sensory-based decision fits into the longer sequence of behavioral steps needed to perform the task. If it is true that the pattern of caudate activity represents a sequence of belief states, then each transition from one distinct pattern to another may correspond to a “decision”, even if the design of the experiment is mostly concerned with the particular state transition that is linked to the animal’s overt choice. This interpretation provides an answer to a long-standing question about the accumulator model—namely, what determines when the accumulation process should start. From the viewpoint of the sequence of behavioral states, the accumulation process would start with the “pre-choice” state that immediately precedes the “choice” state. The transition to this “pre-choice” state, presumably guided by other sensory events and the learned temporal structure of the task, is itself a type of decision, albeit a covert one. For example, in our case, we observed ramp-like activity similar that observed by to Ding and Gold [5], but it began during the delay period prior to the abrupt motion-direction change (Fig 2), suggesting that this was not an accumulation of sensory evidence but a gradual change in belief based on elapsed time [46], urgency [47], or other information.

### Conclusions

Our results provide new insights into the functions of the basal ganglia and how they may contribute to selective visual attention. We found that neurons in the primate caudate nucleus strongly select the spatial location of behaviorally relevant stimulus during specific visual contexts, even in the absence of any overt motor response, and that caudate neuron activity is sufficient to identify the epochs of a covert attention task. These results demonstrate that the role of the basal ganglia in selection extends to nonmotor aspects of behavior and illustrate how the tracking of behavioral states by striatum and related circuits could support nonmotor, cognitive functions such as attention and decision-making, as well as motor functions such as action selection.

## Supporting information

S1 Fig Summary of joystick movements in different task conditions. (A) Mean joystick voltage traces for hits when the spatial cue was located at the contralateral location (orange) or at the ipsilateral location (blue) for Monkey R (left panel) and Monkey P (right panel) aligned from joystick release. Dashed lines indicate the average trace, whereas shaded areas represent 95% CI (mean +/− standard deviation). The dashed horizontal lines indicate the joystick voltage threshold for releasing (2 V), and the dashed vertical lines indicate the time marked as joystick releases. (B) Joystick voltage traces for hits for CD task (black) and hits for single-patch conditions (red). (C) Joystick voltage traces for hits for CD task (black) and for hits for the joystick task (blue). Contralateral and ipsilateral trials were pooled for panels B and C. Same conventions as (A) for panels B and C. CD, change-detection.
(EPS)

S2 Fig Categorization of caudate neurons using alternate procedures for ranking based on peak activity. (A) Normalized activity for the complete population of caudate neurons (*n* = 227), ranked according to the time of their peak activity for contralateral cue conditions. Each row represents the normalized activity of a single neuron for contralateral presentation of the spatial cue (left) or ipsilateral (right) aligned on the motion stimuli onset. Solid white lines indicate onset of motion stimuli onset; dashed lines indicate spatial cue onset. (B) Same conventions as in (A) except that neurons were ranked according to the time of their peak activity for ipsilateral cue conditions. (C) Scatterplot of peak times for ipsilateral trials as a function of peak times for contralateral trials. Each dot represents one caudate neuron (*n* = 227). Categories are delimited by dashed lines. Peak times obtained by ranking for contralateral and ipsilateral cue conditions were highly correlated (Pearson correlation, p < 0.001).
(EPS)

S3 Fig Response-choice activity analyzed by aligning on MC onset rather than joystick release. Scatter plot of detect probabilities and sensory AROC values computed for each caudate neuron that showed response-choice activity (*n* = 80) during a 0.3-s period aligned on MC onset. AROC values on the x-axis greater (less) than 0.5 indicate a preference for contralateral (ipsilateral) hits. Each dot represents one caudate neuron (*n* = 80). Color indicates the neuron’s group assignment based on the AROC values: hits versus misses AROC different from chance (“motor choice”, red), contralateral hits versus ipsilateral hits different from chance (“sensory”, blue), both different from chance (“sensorimotor”, green), or neither different from chance (black). AROC, area under the receiver operating characteristic curve; MC, motion change.
(EPS)

S4 Fig Time course of sensory AROC values and detect probability during the visual attention task. (A) Representation of sensory AROC values computed between responses to contralateral trials versus ipsilateral trials. Each row represents the AROC values of a single neuron computed between contralateral and ipsilateral conditions, aligned on motion stimuli onset (left panel; 0.05-s bin width) and on MC (right panel). Neurons were ranked according to the time of their peak activity across both contralateral and ipsilateral cue conditions. Colors indicate significant side preference (orange for contralateral, blue for ipsilateral, gray for neither, AROC > 0.5). (B) Representation of detect probabilities (AROC values) computed between responses to hits versus misses for contralateral event conditions (top row) and ipsilateral event conditions (bottom panel) aligned on motion stimuli onset (left column) and on MC (right column). Colors indicate significant preference for hits or misses (red for hits, black for misses, gray for neither, AROC > 0.5). (C) Summary plots showing percentages of caudate neurons with significant sensory AROC (blue curve) and detect probability (red curve) values at each time point in the attention task. In all panels, dashed lines indicate onset of spatial cue; solid lines indicate onset of motion stimuli and MC. AROC, area under the receiver operating characteristic curve; MC, motion change.
(EPS)

S1 Table Activity of response-choice neurons earlier in the attention task. For all neurons with response-choice activity (*n* = 80), the table indicates the neuronal categories assigned to these neurons based on activity during early epochs of the motion-direction CD task. The rows of the table follow the categories illustrated in Fig 4B; the columns follow the categories shown in Fig 2B. CD, Change detection.
(EPS)

S1 Data Data underlying the paper. (XLSX)

## Abbreviations

AC: anterior commissure
AMI: Attention Modulation Index
AROC: area under the receiver operating characteristic curve
CD: change-detection
LIP: lateral intraparietal area
MC: motion change
MGS: memory-guided saccade
MST: medial superior temporal area
MT: middle temporal area
PAN: phasically active neuron
PSTH: peristimulus time histogram
ROC: receiver operating characteristic
SMI: Side Modulation Index
sp/s: spikes per second
SVM: support vector machine
TAN: tonic active neuron (cholinergic interneuron)
V1: visual area #1
V4: visual area #4
